# Community-Engaged Participatory Methods to Address Lesbian, Gay, Bisexual, Transgender, Queer, and Questioning Young People’s Health Information Needs With a Resource Website: Participatory Design and Development Study

**DOI:** 10.2196/41682

**Published:** 2023-09-07

**Authors:** Daniel Delmonaco, Shannon Li, Christian Paneda, Elliot Popoff, Luna Hughson, Laura Jadwin-Cakmak, Jack Alferio, Christian Stephenson, Angelique Henry, Kiandra Powdhar, Isabella Gierlinger, Gary W Harper, Oliver L Haimson

**Affiliations:** 1 University of Michigan Ann Arbor, MI United States

**Keywords:** lesbian, gay, bisexual, transgender, and queer health, LGBTQ+ health, information seeking, participatory design, community-based research, web-based health resources, lesbian, gay, bisexual, transgender, and queer young people, LGBTQ+ young people, mobile phone

## Abstract

**Background:**

Lesbian, gay, bisexual, transgender, queer, and questioning (LGBTQ+) young people (aged 15 to 25 years) face unique health challenges and often lack resources to adequately address their health information needs related to gender and sexuality. Beyond information access issues, LGBTQ+ young people may need information resources to be designed and organized differently compared with their cisgender and heterosexual peers and, because of identity exploration, may have different information needs related to gender and sexuality than older people.

**Objective:**

The objective of our study was to work with a community partner to develop an inclusive and comprehensive new website to address LGBTQ+ young people’s health information needs. To design this resource website using a community-engaged approach, our objective required working with and incorporating content and design recommendations from young LGBTQ+ participants.

**Methods:**

We conducted interviews (n=17) and participatory design sessions (n=11; total individual participants: n=25) with LGBTQ+ young people to understand their health information needs and elicit design recommendations for the new website. We involved our community partner in all aspects of the research and design process.

**Results:**

We present participants’ desired resources, health topics, and technical website features that can facilitate information seeking for LGBTQ+ young people exploring their sexuality and gender and looking for health resources. We describe how filters can allow people to find information related to intersecting marginalized identities and how dark mode can be a privacy measure to avoid unwanted identity disclosure. We reflect on our design process and situate the website development in previous critical reflections on participatory research with marginalized communities. We suggest recommendations for future LGBTQ+ health websites based on our research and design experiences and final website design, which can enable LGBTQ+ young people to access information, find the right information, and navigate identity disclosure concerns. These design recommendations include filters, a reduced number of links, conscientious choice of graphics, dark mode, and resources tailored to intersecting identities.

**Conclusions:**

Meaningful collaboration with community partners throughout the design process is vital for developing technological resources that meet community needs. We argue for community partner leadership rather than just involvement in community-based research endeavors at the intersection of human-computer interaction and health.

## Introduction

### Background

Lesbian, gay, bisexual, transgender, queer, and questioning (LGBTQ+) young people’s unique health needs are often underserved in the United States by institutional information sources such as school curricula and health care providers [1-3]. However, beyond information access issues, LGBTQ+ young people may need information resources to be designed and organized differently compared with their cisgender and heterosexual peers. In this paper, we describe our community-based participatory approach to developing a new web resource with relevant, comprehensive, and easily navigable health information for LGBTQ+ young people. Our design process and reflections will inform future LGBTQ+ digital health resources and important considerations for health research with other marginalized communities.

LGBTQ+ people face substantial health disparities compared with the general population in the United States [4-6]. In a 2020 national survey of LGBTQ+ youth in the United States, the Trevor Project reported that 40% of LGBTQ+ respondents aged 13 to 24 years seriously considered attempting suicide and 68% reported symptoms of generalized anxiety disorder [6]. Transgender and nonbinary youth reported even higher instances of these mental health indicators [6]. During the COVID-19 pandemic, a study reported that 60% of sampled LGBTQ+ college students experienced some type of psychological distress, anxiety, or depression and nearly half came from unsupportive immediate families [7]. In addition, young LGBTQ+ people face difficulties accessing relevant resources to address their health needs because of homophobia, transphobia, privacy concerns, and fear for safety if outed as a gender or sexual minority [8]. Increased access to information and resources might begin to address some of these health disparities by empowering LGBTQ+ young people with increased knowledge about their own bodies and health needs as well as increased awareness of local LGBTQ+-affirming services and groups. We discuss one such effort in our collaborative design of a new LGBTQ+ health website with a community partner and LGBTQ+ young people.

Young people have different information needs related to gender and sexuality from those of older people as young people are often still exploring and experimenting with their gender and sexual identities and face challenges in keeping information seeking hidden from close family members who may still exercise control over some digital aspects of their lives [9,10]. LGBTQ+ young people in particular require special considerations in health care as they experience high risks of mental health issues, including suicidality and depression, substance abuse, discrimination, violence, and homelessness [11,12]. As a result, we set out to design a web-based health care resource site to support LGBTQ+ young people in their health information needs.

Our partner in this project was the Community Health Access Initiative (CHAI) Action Committee, a group of LGBTQ+ young people. CHAI is a participatory, community-engaged project that includes academic partners, LGBTQ+ young people, and community partners from local youth-serving organizations [13]. CHAI works to improve LGBTQ+ young people’s health by increasing access to LGBTQ+-affirming health care training for local health care providers, with a focus on Washtenaw County in Southeast Michigan. CHAI’s efforts are youth-driven, and the project relies on the insights of its Action Committee, an advisory group of LGBTQ+ young people. This collaborative research and design project emerged from the Action Committee’s desire to create a new CHAI website with updated resources and information to address the health information needs of LGBTQ+ young people, who often struggle to find relevant, trusted information both in person and on the internet. The following research questions (RQs) guided our study:

- RQ1: What health information needs of LGBTQ+ young people are not addressed by existing digital resources?
- RQ2: How might we use participatory methods to meet some of the health information needs of local LGBTQ+ young people with our partner’s new website?
- RQ3: What unique features should future web-based health resources for LGBTQ+ young people include to ensure that LGBTQ+ young people can both access and find the information most relevant to their own identities?

To answer these questions, we conducted 17 interviews and held 3 design sessions (n=11) with LGBTQ+ young people aged 15 to 25 years (n=25; 3/25, 12% of the participants took part in both an interview and design session) to explore their health needs and how the new CHAI website might address their information needs. With this study, we make the following contributions: (1) greater understanding of LGBTQ+ young people’s health information needs and how a web-based resource such as the new CHAI website may address these needs; (2) new design recommendations for LGBTQ+ web-based health information resources, particularly to ensure that LGBTQ+ young people can both access and find information relevant to them; (3) description of and reflection on a community-based participatory design and development process for an LGBTQ+ web-based health resource; and (4) extending Harrington et al’s [14] participatory design tensions with an additional consideration: *community partner leadership* in human-computer interaction (HCI).

We find that LGBTQ+ young people have specific needs for a health information resource site: they must be able to access credible information but also find the right information for themselves, in particular while navigating identity disclosure concerns. We describe several design features that help address these needs. In addition, we find that designing for intersecting marginalized identities is vital—participants in this study described how they could not separate gender from other marginalized identities. Thus, a resource site for LGBTQ+ young people must meaningfully enable LGBTQ+ young people to find resources related to all of their identities, reflecting how those identities intersect. Taken together, our findings suggest that LGBTQ+ young people have specific health concerns, information needs, and design considerations that are often absent from existing web-based and offline resources. Our design work incorporates these into a new web-based resource, and we present our design process for the website and reflect on using participatory methods with LGBTQ+ young people.

Although we recognize that a website is not a particularly novel design intervention in 2023, we nonetheless considered it important to design and develop the type of tool that young LGBTQ+ participants and community partners considered most important for their needs. Thus, although the technology may not be particularly compelling on its own, in this paper we describe design features and content types that reflect LGBTQ+ young people’s health information needs and suggested designs as these information-seeking needs cannot be fully met with existing resources. Thus, in this paper, we describe how we achieved our aim of working with a community partner to design and develop an inclusive and comprehensive new website to address LGBTQ+ young people’s health information needs while identifying and incorporating content and design recommendations from young LGBTQ+ participants.

### Related Work

#### LGBTQ+ Health and Technology

The internet is a vital health information resource for LGBTQ+ young people, especially for health information that they are often unable to receive from health care professionals and in school settings, such as inclusive sex education [1,3,15,16]. Charest et al [2] reported that LGBTQ+ participants relied more on educational websites and news outlets for health information than on traditional health classes. LGBTQ+ and other young people may benefit from technological health interventions such as mobile information tools to access sexual health information [17,18], smoking cessation support [19], peer support [20], and photoelicitation methods to expand the understanding of LGBTQ+ identities [21].

LGBTQ+ young people may prefer digital resources and media because of insufficient school curricula, lack of relevant offline resources, and health care providers’ inability to meet the health needs of LGBTQ+ patients [3,22]. In addition, isolation, stigmatization, and lack of information motivated sexual and gender minority young people to use the internet to meet their information needs [23,24]. However, in digital spaces, LGBTQ+ young people may experience additional barriers, such as privacy concerns, accuracy limitations, and difficulty finding local information [8,25]. These disparities and difficulties motivated our creation of a new CHAI website.

Addressing LGBTQ+ mental health disparities was particularly important for both the CHAI Action Committee and the participants. Previous research has presented possible digital solutions to address some of these concerns. Web-based platforms can be especially important for LGBTQ+ young people to access remote, immediate mental health support, particularly during the COVID-19 pandemic [26,27]. Participants in our project discussed many topics related to mental health and well-being.

Web-based experiences vary greatly within the LGBTQ+ community as different sexual and gender minority groups within this larger group are not monolithic. For example, transgender and nonbinary individuals have specific safety concerns in digital spaces [25,28,29]. Matsuno et al [30] recommended developing “subpopulation-specific interventions” to account for the unique difficulties each group faces. As we will present in our results, participants recognized the need for health information that accounts for multiple, intersecting marginalized identities. Although a website will not solve the pervasive structural disparities faced by LGBTQ+ young people in health care, our participatory approach and creation of the CHAI website will provide easily accessible, relevant, and inclusive health information tailored to LGBTQ+ young people.

#### Participatory Methods

Our approach for designing and developing the CHAI website is situated in community-based participatory design because of the emphasis on including participants with marginalized identities and collaboration with community entities. Participatory design is a natural fit for community-driven work as it facilitates two important components: (1) people affected by designs should have a say in the design process and (2) communities adopting a technology should be included to ensure that the final design meets their needs [31]. We aim to meet these goals by including the intended audience, LGBTQ+ young people, in designing the CHAI website and in our research process. Merkel et al [32] argued that participatory design in computing involves community members’ leadership in the design process and the direction and maintenance of design outcomes. CHAI and the Action Committee directed our design process and helped ensure that the effects and outcomes could be sustained. In addition, participatory methods facilitate project goals geared toward improving marginalized experiences through technology. Unertl et al [33] argued that community-based participatory research is particularly beneficial both in engaging populations traditionally underserved by health care systems and in designing patient-facing technologies to address health needs. In the CHAI website development, we used participatory techniques to design a new site with and for LGBTQ+ young people.

Researchers have discussed important considerations when engaging marginalized populations using participatory methods [14,34-38], which can improve engagement with intended communities and better meet digital project goals. In developing our participatory methods, we sought to address these considerations with our participants and community partner. Costanza-Chock [37] presented the design justice framework, developed in collaboration with the Design Justice Network, and urged designers to consider the values we encode and reproduce through design, who is involved in the design process, how design is framed, and where we design. The CHAI project’s design incorporated these considerations as we centered the Action Committee and other LGBTQ+ young people throughout the project. We sought to demystify the design process for participants and incorporate LGBTQ+ young people and the community partner at each step, from inception to deployment. We also carefully considered participants’ intersecting identities and future website users. Young people in particular are often excluded from design processes and, thus, may be further marginalized in technological systems [39].

Participatory research with LGBTQ+ participants [40-46] has helped align technology design with community needs and provides important takeaways for this project. Sugarman et al [47] used participatory methods with LGBTQ+ racial and ethnic minority groups in health contexts and recommended the importance of cultural humility when working with marginalized populations and community partners. Benjamin [48] discussed the importance and value of speculative methods, especially for marginalized groups, as a way to envision new solutions and possibilities. In this project, although we did not constrain participants to practical approaches, primarily practical design ideas arose in design sessions and, thus, became the technical features implemented in the website design.

## Methods

**Figure 1:**
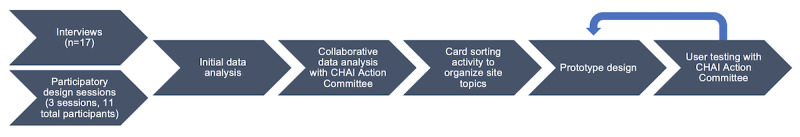
Research and design method workflow. CHAI: Community Health Access Initiative.

To design a new web-based LGBTQ+ health resource website driven by community needs, our methods included interviews, participatory design sessions, collaborative data analysis, card sorting, iterative prototype design, and user testing, as shown in Figure 1. In conducting our research and preparing this manuscript, we referred to several applicable reporting guideline checklists: the iCHECK-DH (Guidelines and Checklist for the Reporting on Digital Health Implementations) [49] and the Standards for Reporting Qualitative Research (SRQR) [50].

### Data Collection

We conducted interviews with 17 self-identified LGBTQ+ young people aged 15 to 25 years and held 3 web-based design sessions with 11 self-identified LGBTQ+ young people aged 16 to 24 years. In total, 12% (3/25) of the participants took part in both; thus, our total sample size was 25. This age range reflects the ages of the young people that CHAI serves. The interviews and design sessions were conducted throughout the same period.

In the interviews, we asked participants about their health information–seeking experiences both on the internet and offline. We recruited 47% (8/17) of the participants for the interviews via recruitment information on the authors’ Twitter accounts and 35% (6/17) via email recruitment distributed by CHAI and our community partner, the Michigan Organization on Adolescent Sexual Health. Finally, we recruited 18% (3/17) of the participants through User Interviews (User Interviews Inc) [51], a participant recruitment service. These mixed recruitment methods enabled us to interview young people in demographic categories not represented in the sample that we recruited with our community partners’ assistance (eg, Black young people). We confirmed recruitment criteria—participants must identify as members of the LGBTQ+ community; be aged 15 to 25 years; and be comfortable reading, writing, and speaking English—using a web-based screening survey. We purposely sampled a diverse group of participants on 4 dimensions: age, gender, sexuality, and race and ethnicity. All interviews were conducted via videoconference and averaged 56 (range 43-74) minutes.

**Figure 2:**
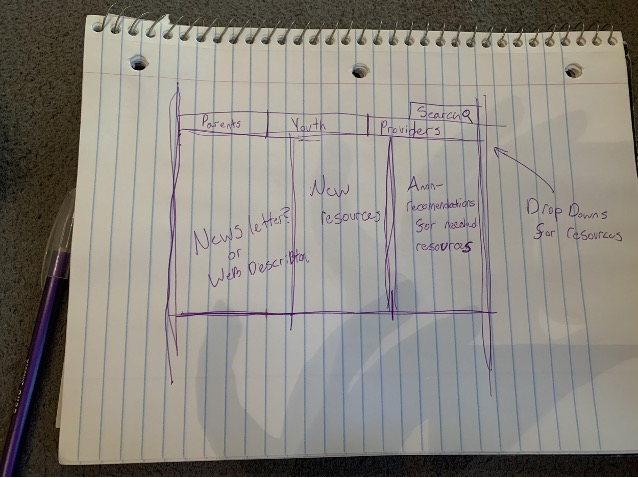
Design sketch from Community Health Access Initiative (CHAI) design session.

**Figure 3:**
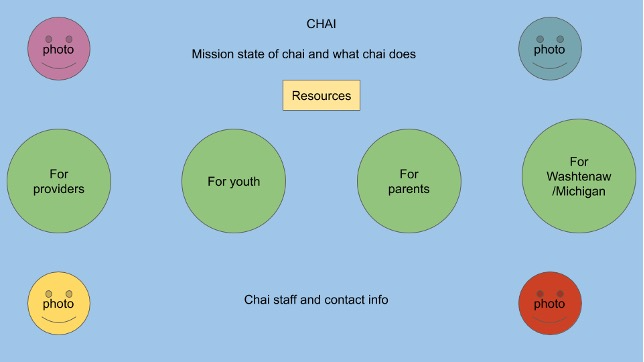
Sample sketch of a design from one of the Community Health Access Initiative (CHAI) design sessions emphasizing photos; different options for parents, youth, and providers; and local resources.

For the design sessions, we recruited participants using recruitment emails and convenience sampling in coordination with our partners. In total, 4 members of the CHAI Action Committee participated in the study. The design sessions began with semistructured interviews in which participants discussed health information–seeking experiences and desired health information. We asked participants to list health topics that they struggled to find relevant information about and brainstormed ways to address these needs. We followed this discussion with design activities in which participants created designs for potential LGBTQ+ web health resources. We prompted participants to draw on how the website might ideally look and any functionalities or tools it might contain. Participants then presented and discussed their designs with each other. Figures 2 and 3 are examples of designs created in participatory design sessions that informed our development process. Each design session included 3 to 4 participants for a total of 11 participants, and the average design session length was 89 (range 85-92) minutes. Each participant took part in only 1 of the 3 sessions.

### Ethics Approval, Informed Consent, and Compensation

This study received approval from the University of Michigan Institutional Review Board (HUM00174029). All participants provided verbal consent after reviewing the institutional review board–approved consent documents. We received a waiver of parental consent for participants aged <18 years so that participation in this project would not need to reveal their LGBTQ+ status to parents or guardians. Participants were reminded that the interviews and design sessions were entirely voluntary and could be stopped at any point. The data were anonymized and deidentified after collection to protect participant confidentiality. We discuss our decisions regarding presenting participant demographics in the Results section and how we try to maintain participant anonymity while presenting the results. For the individual interviews, each participant received a US $25 gift card for taking part. For the design sessions, CHAI Action Committee members received hourly compensation through their existing positions with CHAI (n=4). Design session participants who were not Action Committee members (n=7) received a US $40 gift card for taking part. All participatory design sessions were conducted via videoconference.

### Data Analysis

Our research team met with CHAI and its Action Committee to collaboratively analyze data, discuss emerging results, and present designs throughout the data analysis and design processes. All the design sessions and interviews were recorded and transcribed. The first author completed detailed memoing after each design session and interview to begin to identify themes. The first and second authors qualitatively analyzed the data using initial open coding followed by axial coding [52]. In our data analysis and the development of the new CHAI website, we conducted member checking [53] by (1) including Action Committee members in the research process through collaboratively identifying themes in axial coding processes [52] and opportunities to provide feedback on this paper, (2) user testing website iterations via feedback sessions and task analysis with Action Committee members and discussions with them and CHAI leadership at their regularly scheduled meetings, and (3) including interview and design session participants and Action Committee members in the card sorting activity to identify and group the most important health themes from the interviews.

After coding, community members, including Action Committee members, design session participants, and CHAI leadership, grouped these codes into themes in a meeting led by the first author. All Action Committee members identify as LGBTQ+. The Action Committee and CHAI leadership used Miro (RealtimeBoard, Inc) [54], a web-based collaborative tool, to group qualitative codes produced by the first and second authors into relevant themes. Community members discussed their thinking throughout the process to reach an agreement on the themes. These themes include the sections we focus on in this paper, such as resource topics to include on the website, LGBTQ+ stories and experiences, intersectional LGBTQ+ health experiences, technical website features, and recommendations for future LGBTQ+ digital health resources.

### Design Process

Once we identified potential website topics, we conducted a card sorting activity with participants to organize the health topics mentioned in the design sessions and interviews and determine how they should be organized on the new website. Card sorting is a method that enables researchers to understand how people categorize concepts [55]. We used Optimal Workshop (Optimal Workshop Ltd) [56], a web-based tool, for the card sorting tasks. In the Results section, we present the results of the card sorting activity, which directly informed the website’s organization.

**Figure 4:**
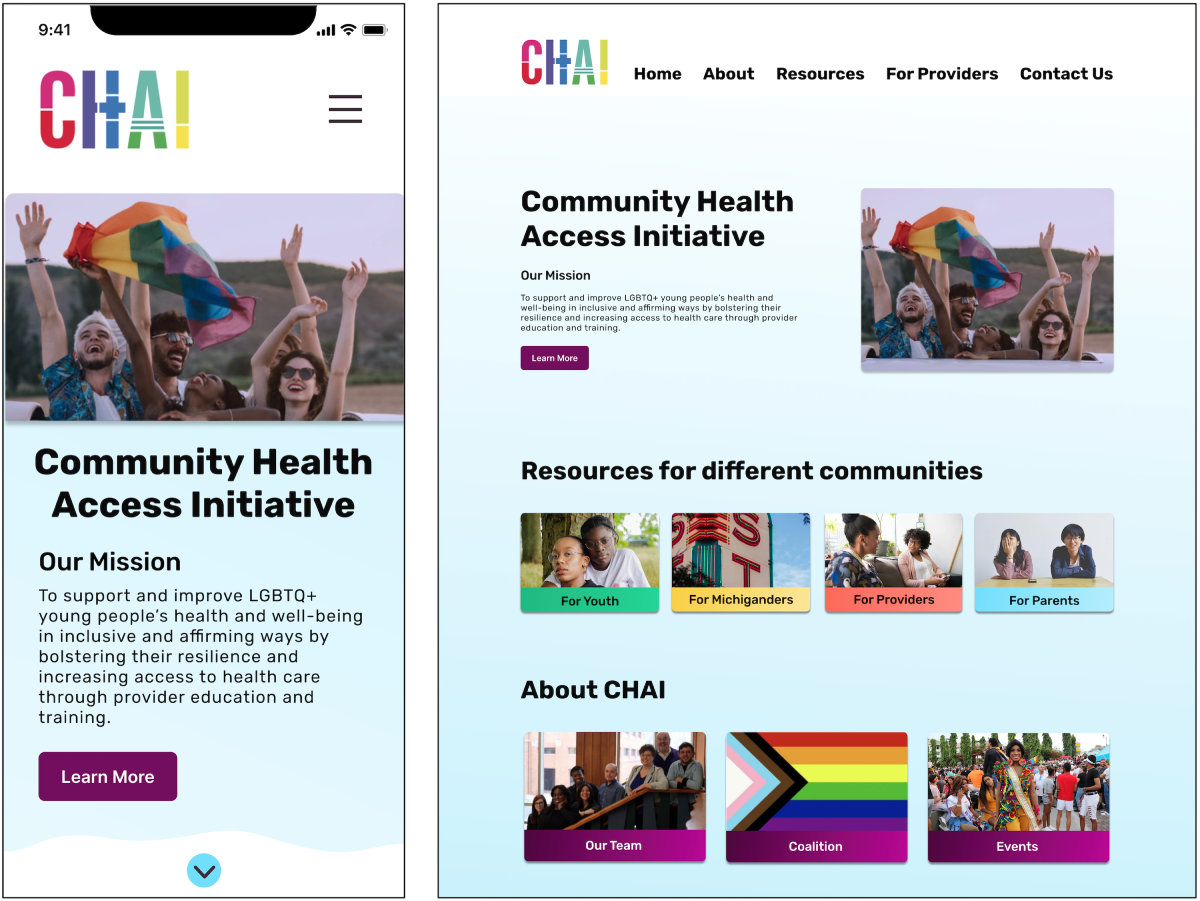
A prototype of the new Community Health Access Initiative (CHAI) website based on designs and suggestions from interviews and participatory design sessions. Mobile (left) and web (right).

The third author designed the website using a human-centered design process [57] in which LGBTQ+ young people (the Action Committee) were involved from the beginning; developed initial design ideas (in the design sessions); and gave feedback frequently (via feedback sessions and written correspondence), which we incorporated into the site design. Action Committee members received hourly compensation for this work through their existing positions with CHAI. Our iterative design process included regular team meetings with CHAI, frequent feedback, and user testing with design session participants. We conducted two 30- to 45-minute rounds of feedback and user testing sessions with the 5 Action Committee members. User testing [58] included completion of tasks such as finding specific resources on different versions of the new website. Action Committee members regularly provided qualitative feedback during user testing, including positive reception of both the mobile and desktop versions of the redesigned website (see the Results section). Figure 4 shows the final digital prototype that was launched after the study was completed.

## Results

In this section, we will describe our results, which achieve our aim of identifying and incorporating content and design recommendations from young LGBTQ+ participants to design a new web-based LGBTQ+ health information website in partnership with the community.

### Participant Demographics

Participant demographics are presented in Table 1 at the aggregate level to protect participants’ anonymity. All the participants lived in the United States at the time of taking part in the study. We do not include more specific information about race and ethnicity or age for specific participants to maintain participant anonymity.

**Table 1:** Participant demographics (n=25).

Characteristic			Values
**Gender, n (%)**			
	Cisgender woman	11 (44)	
	Nonbinary	6 (24)	
	Transgender man	3 (12)	
	Cisgender man	2 (8)	
	Transgender woman	1 (4)	
	Genderqueer	1 (4)	
	Questioning	1 (4)	
**Sexuality, n (%)**			
	Bisexual	8 (32)	
	Pansexual	5 (20)	
	Queer	5 (20)	
	Gay	2 (8)	
	Questioning	1 (4)	
	Polysexual	1 (4)	
	Biromantic asexual	1 (4)	
	Heteroromantic grey asexual	1 (4)	
	Lesbian	1 (4)	
**Race and ethnicity, n (%)**			
	American Indian	1 (4)	
	Asian or Asian American	4 (16)	
	Black or African American	3 (12)	
	Latinx	1 (4)	
	Middle Eastern and North African	2 (8)	
	Multiracial or biracial	5 (20)	
	White	9 (36)	
**Education level, n (%)**			
	Some college	9 (36)	
	College graduate	7 (28)	
	Some high school	6 (24)	
	Some graduate school	2 (8)	
	High school graduate	1 (4)	
Age (years), mean (SD; range)			20 (3; 15-25)

### What Young LGBTQ+ Participants Did Want

In this section, we identify both the topics and features that participants described wanting the new CHAI website to include. This section draws from our qualitative coding processes, as described in the Data Analysis section.

#### Resource Topics to Include on the Site

Interview and design session participants detailed numerous desired health resources and topics to be included on the CHAI website. Using card sorting, participants then grouped and prioritized these findings into 10 main resource topics with subtopics, as detailed in Textbox 1 [55]. Topics and subtopics directly informed the website’s organization and signaled the areas where information might not be available elsewhere. Many topics dealt with difficulties accessing inclusive care or information. Participants also expressed a desire for information for health care providers and parents to best support LGBTQ+ young people.

**Resource topics to include on the site, based on results from the resource topic card sorting activity.**

**Relationships**

- Consent
- Relationship violence
- Internet and app-based dating
- Sexual assault and sexual harassment

**Sexual and reproductive health**

- Menstruation
- Testing (HIV, sexually transmitted infections, and pregnancy)
- Sexual protection information
- Lesbian, gay, bisexual, transgender, queer, and questioning (LGBTQ+)–inclusive sexual education
- Sexual assault and sexual harassment

**Navigating health care and finding a provider**

- Health screening
- Changing health care providers
- Knowing your medical rights
- Navigating the insurance system
- Finding LGBTQ+-inclusive providers
- Health care confidentiality
- How to deal with physicians that are noninclusive
- Health care provider reviews and recommendations from LGBTQ+ people
- Navigating finances
- Finding a therapist

**Navigating identity and coming out**

- Coming out
- Resources for intersecting identities
- Navigating sexual and gender identity
- Resources specifically for LGBTQ+ young people
- Finding community

**LGBTQ+ 101**

- Terms related to LGBTQ+ (glossary)
- LGBTQ+ history
- Pronouns
- Debunking misinformation about LGBTQ+ people
- LGBTQ+ people’s personal success stories

**Transgender and nonbinary resources**

- Gender transition resources
- Using binders
- Gender dysphoria
- Hormone injection how-to
- Transgender people’s inclusion in sports
- Affirmative care specifically for transgender and nonbinary people
- Inclusive restrooms in health care facilities

**Crisis resources**

- Sexual assault and sexual harassment
- Homelessness
- Hotlines
- Navigating social services programs

**Finding community**

- Local community resources
- Navigating social services programs
- Homelessness

**Mental health**

- Mental health resources
- Self-care
- Substance abuse
- Finding a therapist
- Hotlines

**Resources for providers**

- Health screening
- Affirmative care specifically for transgender and nonbinary people
- Inclusive restrooms in health care facilities
- Clinical skills for providers serving LGBTQ+ people
- Inclusive intake forms for LGBTQ+ people
- Resources for providers serving LGBTQ+ people
- Service referrals

**Resources for parents**

- Resources for parents of LGBTQ+ people
- Debunking misinformation about LGBTQ+ people

In the following sections, we present participant suggestions related to 3 themes CHAI Action Committee members identified as part of our collaborative data analysis and member-checking process [53]: *LGBTQ+ stories and experiences, intersectional LGBTQ+ health experiences*, and *technical website features*.

#### LGBTQ+ Stories and Experiences

Participants expressed a preference for LGBTQ+ health resources from people with shared identities and experiences. They argued that this would lead to increased trust in the health information and further connection with other people. P7, a pansexual woman of color, mentioned that, when choosing a health care provider, she preferred “relying on personal accounts of people’s experiences. I want to make sure that this person is coming from a personal experience where they believe similar things that I believe*.*” P7 said that such stories helped her understand and feel less uncertain about particular health resources. Many participants stated that, when they heard from trusted people about experiences at a specific provider or clinic, they felt more inclined to use that provider or service rather than unverified services.

P11, a polysexual woman of color who had limited resources and felt isolated as a teenager, turned to digital spaces for videos of LGBTQ+ people describing their experiences and also found necessary health information through videos. P11 stated the following:

YouTube helped me figure out, “Oh, this person labels themselves as this, and they think about it this way. And I think that I’m similar, so maybe I should try out this label.” And then I worked from there and just tried to figure it out for myself. So I think personal accounts really helped me.

Participants’ sentiments informed our design of the website as both an affirming space for those who did not have access to other LGBTQ+ people and a space that might help those seeking out LGBTQ+ health information for the first time. When designing the CHAI website, we considered ways to incorporate LGBTQ+ people’s personal stories and experiences into the site. However, within the scope and timeline of the project, we were unfortunately not able to include such stories.

#### Intersectional LGBTQ+ Health Experiences

In the collaborative thematic grouping process to identify themes related to qualitative codes, Action Committee members identified *intersectionality* as an important theme emerging in participant responses, which informed the content and resources available on the new CHAI website. Many participants in interviews and design sessions described difficulty finding relevant resources that accounted for both their gender and sexuality and other intersecting marginalized identities, including race and disability. Action Committee members reviewed topics related to overlapping marginalized identities and chose to label these data points as “intersectional LGBTQ+ health experiences.” In this section, we present participant information needs related to intersectional aspects of their identities.

Crenshaw [59] introduced intersectionality as a framework for highlighting the systemic oppression and erasure faced by Black women when gender and race are treated as legally disparate categories. Crenshaw’s [59] work builds on a rich critical history of scholarship by other Black women, particularly the Combahee River Collective’s 1977 statement [60], which identified the interlocking systems of oppression experienced by Black women. HCI scholars Rankin and Thomas [61] argued the following: “Intersectionality invites us to think deeply about for whom we design technology, the implications of deploying this technology in the world, and who it advantages or disadvantages.” In designing the CHAI website, we considered how we might explicitly incorporate intersectionality, race in particular, as called for by Rankin and Thomas [61], Erete et al [62], and Ogbonnaya-Ogburu et al [63], to meet users’ intersectional health information needs. Participants with multiple marginalized identities described a lack of representation in existing health resources and called for resources that recognize these intersections, echoing previous research in which LGBTQ+ people with additional marginalized identities felt erased in digital spaces [64]. Participants in our study similarly shared their experiences of feeling that their intersectional health needs were ignored. P21, a queer Arab Muslim woman, said the following:

I think something that would have been really helpful for me to see would be discussions around racial or religious identity and sexual and gender identity.

P21 emphasized facing difficulty when others recognized only one aspect of their identity rather than multiple intersecting identities.

In addition to racism and religious stigma, factors such as socioeconomic status add further barriers to health care access. LGBTQ+ young people might face additional socioeconomic barriers if they need to navigate health resources without using parental insurance. For example, P9, a bisexual woman of color, suggested the following:

Make sure at least in every list [of resources] there is one free or low cost option. Because people might be like I was in my situation, which was I had health insurance, but I didn’t want to go because I was afraid it would get billed, and my parents would have a lot of uncomfortable questions, shaming questions for me that I didn’t want to have to answer.

Without considering how factors such as socioeconomic status intersect with LGBTQ+ identity, resource sites for LGBTQ+ young people could inadvertently reinforce inequitable access. An intersectional approach to resource development is vital throughout the research process as such facets cannot be separated from LGBTQ+ identity when developing and implementing health resources.

#### Technical Website Features

##### Overview

LGBTQ+ young people face several hurdles regarding web-based health resources: accessing information, finding the right information, and navigating identity disclosure concerns. The CHAI site itself addresses the first hurdle by making resources available. In this section, we describe 2 features (filters and search) that help LGBTQ+ young people find the right information on the site and 1 feature (dark mode) that helps with navigating identity disclosure concerns. Taken together, these results demonstrate how LGBTQ+ young people have not only unique health information needs [1,2,65,66] but also unique needs in how that information is presented and organized. Participants also emphasized basic web accessibility needs, but we do not discuss that in this section as the topic has been covered at length in prior work.

##### Filters

The CHAI website includes many LGBTQ+ health resources, and for each person, some of these will be relevant and some will not depending on identity facets. Thus, participants suggested filters as an organizational mechanism to refine the results. Filters would allow users to search for information specifically related to their identities or desired topics. Participants suggested enabling users to filter the site into 3 main areas for different audiences; in the words of P8 (a nonbinary and pansexual person of color), “One resource drop down for parents, one for youth, and one for providers.” Owing to the numerous and varied resources that may surface in search results, filtering via categories related to sexuality, gender, and types of information can help narrow down a search. During the design session, P8 shared ideas for filtering and categorizing resources:

I put “mental health resources,” or if you’re looking for more information about basically LGBTQ identities with searching “questioning”—separating it into the more chunky categories and allowing people to say, “Okay, so if I want to find mental health resources, I go here, but if I wanted help navigating healthcare or insurance for mental health, maybe I could go to ‘navigating healthcare’ and that would also pop up.”

As P8 described, topic categories can help narrow down resources so that they align with information needs, making information accessible and easy to find. For instance, a person may be experiencing depression but may not know that *mental health* would be the correct search term to find what they are looking for; filters help in this case.

Beyond filtering by topic categories, participants described wanting to filter based on the user’s identities. P22, a White and bisexual transgender man, described a filtering approach related to identity facets:

I thought it would be helpful to organize the resources by who they were for.

Identity can be an especially helpful way to narrow down resources; for instance, some are primarily for lesbians, people of color, or transgender people. In addition, a filter mechanism that allows people to select multiple identity categories enables filtering based on certain overlapping identities. In the future, we hope that sites such as CHAI can add more information about and features to explore how additional identities intersect with LGBTQ+ health information and update filters to reflect these additions.

In our final version of the CHAI site, filters allow participants to narrow down information by target audience, health topic, and source of the health information. Incorporating filters with the search on the CHAI website can effectively match users with resources to meet their specific health needs.

##### Search

In addition to filters, the search functionality helps users find the resources most relevant to them. Participants expressed a need for improved search functions that were not present in the original CHAI site, such as an easily accessible search bar. Although search is not a novel design feature, it came up in so many of the participants’ designs and quotes that we felt it was important to include it. In describing one of their designs, P8, a nonbinary and pansexual person of color, said the following:

At the top I just have a search engine for if you want to look up one center real quick, if you already know what you’re looking for, instead of going through all those other avenues.

P8’s design (Figure 2) includes this search bar at the top of the resource page.

##### Dark Mode

Many sites and applications offer “dark mode” user interfaces that claim to reduce eye strain by decreasing screen brightness. However, in our interviews, we somewhat unexpectedly learned that dark mode can serve a purpose beyond its visual benefits to potentially help LGBTQ+ young people manage identity disclosure. In a user testing session, an Action Committee member described their experiences browsing the CHAI website:

If I was in the closet and I was trying to look up resources on CHAI, but my family was over my shoulder, this brings a lot of attention to that. So maybe having an incognito mode for CHAI or trying to reverse the colors, like how they do it [on Twitter]. Twitter’s got a dark mode.

For LGBTQ+ young people, simply browsing through LGBTQ+ websites may reveal information that they do not want everyone around them to know about. This is especially salient for people who are not yet “out” to their family and do not want their family to inadvertently see LGBTQ+ content they are browsing or searching for. Dark mode and facilitating privacy may be especially important during the COVID-19 pandemic, when many LGBTQ+ young people spend more time at home and in potentially unsupportive environments [27].

Participants mentioned their experiences using dark mode on other social media platforms to demonstrate how this might work on the CHAI website. An Action Committee member said the following:

Instagram has a white page background...and you can change it to a dark mode. It just turns blue and black. So then that way, it doesn’t...stand out to you, or stand out to others as much. So that is good, because white brings your attention to things and so if I was looking up resources and I was not out to anyone, it’s just easy...to make sure no one else’s attention is being brought to my screen.

Dark mode would reduce brightness to make the content on the screen less visible to those surrounding the user, thus making it less likely to reveal their LGBTQ+ identity. During user testing, multiple Action Committee members responded positively to the dark mode feature that we included. We note that some LGBTQ+ sites and apps use a “quick exit” feature that allows the user to quickly exit the site or switch to a different interface for similar purposes. However, a quick exit did not come up in our data collection, so we focused instead on dark mode for information disclosure concerns.

### What Young LGBTQ+ Participants Did Not Want

In addition to identifying what type of environment and features participants did want on the CHAI website, it was also important to understand what they did not want. This section draws from our qualitative coding processes, as described in the Data Analysis section. We briefly describe several elements that participants especially mentioned not wanting on the CHAI site, including an overwhelming number of resources, unreliable or questionable resources, and irrelevant or inappropriate graphics.

On a resource-based site such as CHAI, providing too many resources may make it more difficult and confusing for users to find what is most useful to their specific needs. The more options available, the longer it takes for users to make a decision, which will likely overload the user [67]. According to P22, a White and bisexual transgender man, “in some cases there definitely can be too much, and in other cases with something slightly less common that’s not out there, information about especially trans people and sex is just nonexistent, or at least not easy to access.” A site such as CHAI must find the right balance in providing enough information that is inaccessible elsewhere on the web without providing extraneous information. To overcome this barrier, our CHAI website design narrows down the decision-making process for users by curating credible resources, grouping them based on different categories, and providing a filtering system that gives the users autonomy to find resources that best suit their needs.

Next, when searching for health information on the web, participants faced difficulties determining which information was credible and reliable, echoing previous research [25,66]. When P20, a White asexual person currently questioning their gender, searched for information about sexually transmitted infection testing on Google, they “wanted to also fact check sources, because a lot of the websites I was clicking on weren’t always official websites. So checking from several sources to get an idea of like, ‘Okay. How regular should you get tested?’” Uncertainty with many sources found on the internet led to skepticism and uncertainty about important health concerns. P22 similarly described the following:

Combing through multiple websites since sometimes it says, “Oh, well, this might be what you have. There’s also these other diseases or illnesses that have similar symptoms.”

Some participants tried to find ways to determine which information was most credible, such as “an official domain like .gov or .edu” (P19). In the CHAI redesign, we attempted to address these concerns with well-researched curated resources and links to trusted and credible external health care resources.

Finally, ensuring that images and graphics are both informative and meaningfully targeted to LGBTQ+ populations is crucial. Some image choices may not communicate the intended message and could prevent users from accessing relevant information. For instance, P12, a White bisexual woman, said that some images “are definitely targeted at heterosexual people,” whereas others seemed irrelevant:

...like a picture of a papaya. I’m like, “I’m just trying to learn if I have a UTI or not, I don’t need to see a picture of a papaya.” Just very weird things like that, where it just almost feels like, “Let’s turn it into something cute” instead of, “Here’s an educational resource that’s really easy and accessible to look at.”

P12 demonstrates the importance of a site like CHAI including graphics that are intentionally informative and relevant rather than “cute,” which can distract from the information provided.

### What We Designed

In this section, we describe the design process and present how we addressed participant comments during the development of the new CHAI website (Figure 4). This section builds on the previous sections and draws from the methods described in the Design Process section.

#### Improving on the Existing CHAI Site

**Figure 5:**
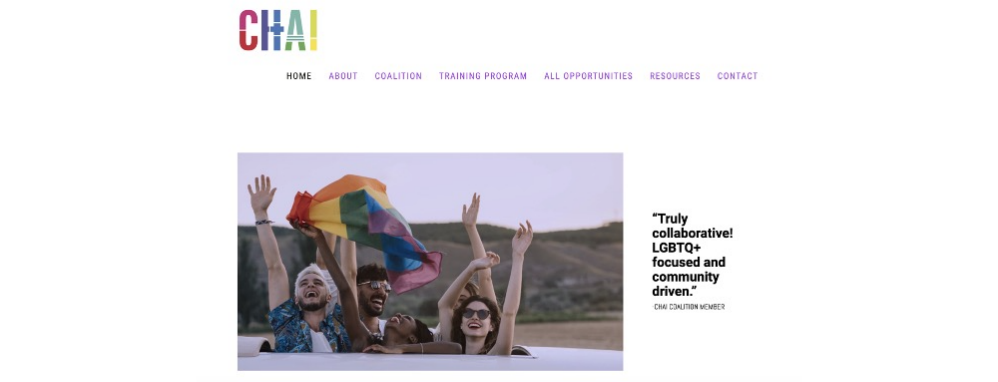
Screenshot of the previous Community Health Access Initiative (CHAI) website.

Before our redesign, we identified aspects of the previous CHAI site (Figure 5) that could be improved and informed by our conversations with participants. These specific concerns arose from CHAI leadership and the Action Committee’s reviews of the earlier site. First, the previous site’s resource section was broad and included a wide range of resources without options for users to narrow down their search. Although the resources were categorized by CHAI on the back end, the site did not allow users enough autonomy to find resources most relevant to them. In addition, the available resources were somewhat limited and may not have met the needs of LGBTQ+ people with multiple marginalized identities. Some of the site’s tabs, such as “All Opportunities” and “Resources,” led the user to broad, yet also limited, collections of content. For example, the “Resources” page included several useful PDF resources but did not entirely capture the complex, multifaceted nature of health care resources LGBTQ+ young people need. Taken together, the existing CHAI site limited users’ ability to personalize their search for information. Our site redesign considers what we learned in our research and increases users’ ability to find information and resources tailored to their identities and health care needs.

#### Site Organization and Information Architecture

As part of our design sessions, participants drew on how they envisioned that the website might be organized. In participants’ sketches, most separated content based on categories such as identities, site audiences, and geographical locations (Figures 2 and 3). The categorization ideas that participants provided helped inform how we could organize the site to best match users’ mental models and enable users to search for information. Participants’ drawings informed what aspects we included on the site’s home page (Figure 4), whereas our card sorting activity showed user preferences and priorities for how to organize the site’s resources into categories.

#### Site Features

The content of the new CHAI website expanded on the resources and functions of the previous CHAI website. Drawing on our findings, the resources are both searchable and filterable. CHAI leadership and the Action Committee researched and designed resources for the website based on the information needs expressed by participants in the interviews and design sessions. As LGBTQ+ public health experts, the CHAI leadership advised the Action Committee in developing specific resources and how to categorize them for filtering. External resource links on the site were vetted by CHAI leadership for credibility and accuracy.

**Figure 6:**
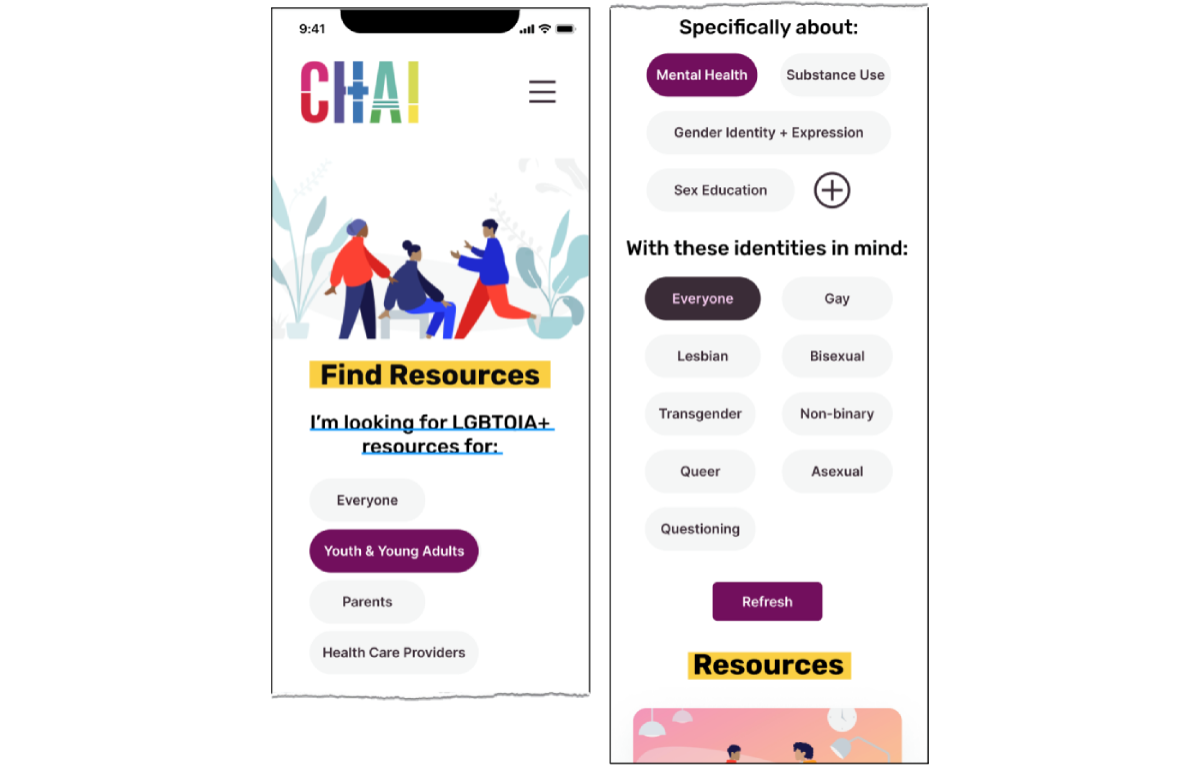
Mock-ups of filtering features for the new Community Health Access Initiative (CHAI) website.

**Figure 7:**
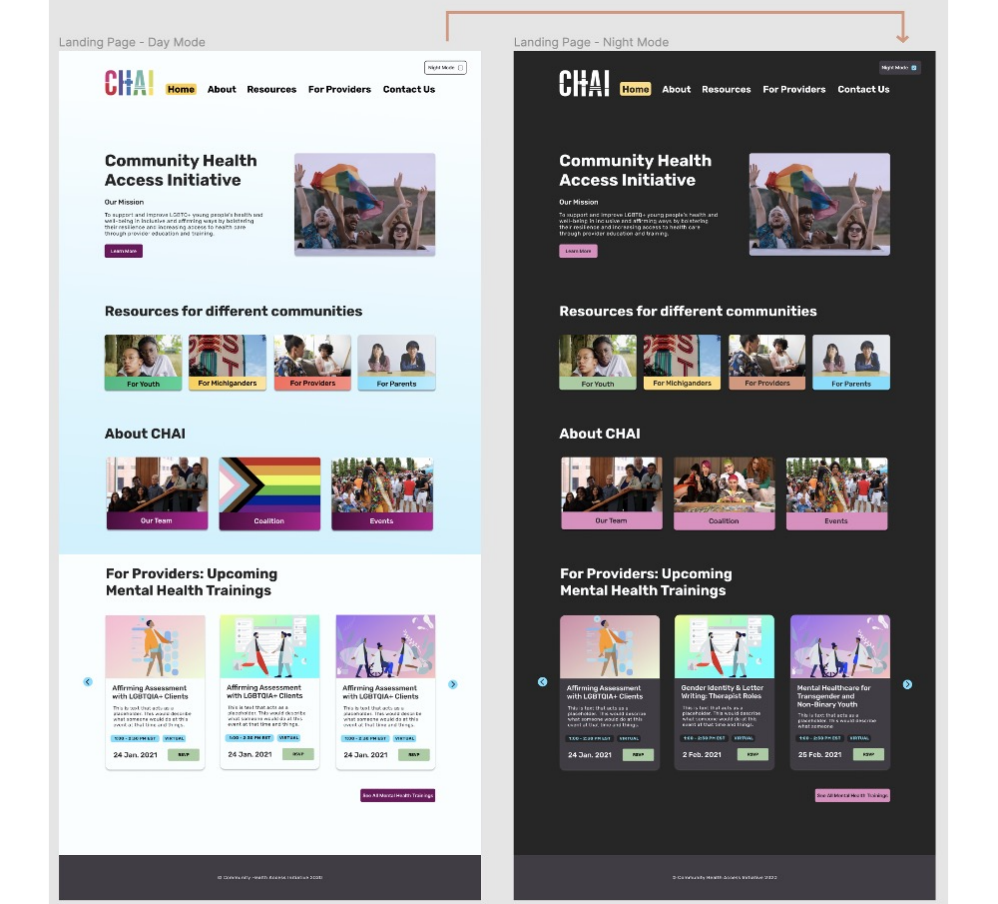
The new Community Health Access Initiative (CHAI) website home page in light mode (left) and dark mode (right).

Findings from the design sessions and interviews, accessible design practices [68,69], and several rounds of user testing with the Action Committee informed design choices in the redesign. Participants’ designs and feedback in user testing gave us insights into what types of information users wanted to see on the site and what features were most important to them—for example, filters, search, and dark mode. Almost all designs from the design sessions included some type of filtering mechanism, so we prioritized incorporating filters. See Figure 6 for an example of the filtering function. Most participants wanted a search function, so we built in search functionality. We prioritized “dark mode” after several Action Committee members noted its potential for protecting privacy and safety. See Figure 7 for an example of the “dark mode” feature. Although dark mode is a good start for a less obviously LGBTQ+ site, to further protect users’ safety and privacy, future iterations of the dark mode feature could swap out rainbow flag pictures and visibly queer content with alternative images.

Importantly, we also strived to incorporate participants’ calls for design and resources that spoke to LGBTQ+ identities’ intersections with race, ethnicity, disability status, and more. Filtering on the site enables participants to select multiple options and, thus, find resources addressing multiple aspects of their identity. In addition, we made intentional image and graphics decisions. Figure 7 shows the images chosen to incorporate a variety of genders, sexualities, races, ethnicities, sizes, and abilities. Finally, we incorporated the overlapping of LGBTQ+ experiences with other identities into both the website’s design and content. We added resources that address health needs arising from additional marginalized identities that intersect with LGBTQ+ identity rather than isolating gender or sexual identity.

Including firsthand accounts of LGBTQ+ people’s health care experiences reflecting overlapping identities would have required a concerted effort to collect those stories or build in a mechanism for people to submit their stories. Although we discussed these possibilities with CHAI and the Action Committee, ultimately, as a group, we decided that social media was already filling this role and that the CHAI site should focus on health resources. Future work may examine how to design a site specifically for sharing intersectional LGBTQ+ health experiences.

### What Young LGBTQ+ Participants Thought

User testing and feedback sessions with the CHAI Action Committee produced vital qualitative feedback throughout the design process and afterward as an evaluative process. This section builds on the previous sections and draws on the methods described in the Design Process section. Some Action Committee members did not participate in interviews or design sessions but did participate in user testing and feedback sessions throughout the web development process. To protect anonymity, we refer to each participant as an Action Committee member in this section without further identifying information.

Overall, Action Committee members responded favorably to the new website. An Action Committee member responded the following:

I think it is really nice, like the setup and everything. It’s not super cluttered with other things. Overall, it’s an easy to navigate website.

This feedback is consistent with our efforts to ensure that our final design was responsive to what participants *do not want*, such as overwhelming amounts of information. Action Committee members also appreciated the mobile capabilities of the website. A member responded the following:

I do like the mobile version, I think that everything is like really nice and bright.

In total, 2 Action Committee members commented that “I like the aesthetic of it a lot” and “I like everything. I love the illustrations.” Careful consideration of the graphics and overall design of the resource page satisfied the expectations and desires of the Action Committee members. Receiving positive feedback on the mobile version is important as some users may need to access it with a smartphone to protect their privacy.

Action Committee members saw particular improvement in the new “Resources” section, emphasizing the new presentation and topic coverage. A member said the following:

I like how [the filter] goes into different categories. I love the format.... And even the subcategories like sex, sex education, mental health.

The Action Committee members valued the range, depth, credibility, and intersectional nature of the new resources and particularly appreciated the filter and search capabilities.

In addition, CHAI provides training to local health care providers. Thus, the new website includes a separate page for providers, with information about training and other provider-facing resources for working with LGBTQ+ young patients. An Action Committee member commented the following:

I like [the provider training page]. I like that. It explains the registration process. And technical assistance is included in that. I think this is helpful.

Additions such as this improved and dedicated “Providers” section came from design sessions with participants familiar with CHAI’s goals. Soliciting feedback from Action Committee members, most of whom participated in the design sessions or interviews, ensured that the final redesigned website met their needs and was consistent with their design suggestions.

## Discussion

### Principal Findings

In this work, we described the results from our interviews and design sessions and our design process for the new CHAI website, which achieves our aim of applying young LGBTQ+ participants’ topic and design recommendations to a web-based health resource for this community. We presented the designs for the new CHAI website and how the design process reflected initial data collection and changed with ongoing feedback from participants and the Action Committee. In this section, we discuss how our work builds on previous research on participatory approaches to design with marginalized populations and provide implications for future participatory design research projects, particularly with marginalized populations. We then present design recommendations for future LGBTQ+ health resources.

We identified important types of desired resources, health topics, and technical website features that can facilitate information seeking for LGBTQ+ young people exploring their sexuality and gender and looking for health resources, including filters that can allow people to find information related to intersecting marginalized identities and dark mode, which can be a privacy measure to avoid unwanted identity disclosure. We found that our participatory approaches to the design process and website development enabled critical reflection on participatory research with marginalized communities. We identified several design recommendations for future LGBTQ+ health websites that will enable LGBTQ+ young people to access information, find the right information, and navigate identity disclosure concerns: filters, reduced number of links, conscientious choice of graphics, dark mode, and resources tailored to intersecting identities.

### Comparison With Prior Work: Participatory Approaches to LGBTQ+ Health Design

#### Overview

Harrington et al [14] presented 4 important tensions for researchers to consider when conducting participatory design with marginalized and underserved populations: the “historical context of the research environment, community access, perceptions of materials and activities, and unintentional harm in collecting full accounts of personal narratives.” Our participatory process and development of the new CHAI website involved each tension, and in this section, we discuss our approaches to and reflections on each in the context of our project. We also present an additional consideration for future participatory research: community partner leadership.

#### Historical Context of the Research Environment

Harrington et al [14] directed researchers conducting participatory work to reflect on the historical context of relationships between researchers and participants. In this research, we accounted for participants’ potential histories dealing with discriminatory or even harmful health care settings and providers because of their status as LGBTQ+ young people [70]. Although not explicitly from a “healthcare setting,” our research team nonetheless might appear to participants as representative of these discriminatory institutional systems, especially because of the university’s close ties with its health system. Power differentials present in the design sessions affect engagement between participants and researchers [71]. To help mitigate these tensions, research team members who identify as LGBTQ+ led all the interviews, design sessions, and other activities.

Incorporating participant and partner organization feedback throughout the research and design processes and involving Action Committee members in the research process informed the final website design and brought community members into the research process. Relationships with CHAI and the Action Committee also led to considerations related to the design justice framework by Costanza-Chock [37], particularly how the community’s intersecting oppressions would be affected by the design process and the website’s design. We aimed for interviews, design sessions, and the website development process to account for not only participants’ and future users’ LGBTQ+ identities but also other identities that would affect participation and future website use.

We relied on frequent communication with the CHAI team to understand concerns and other feedback that arose over the course of the project. This communication strategy helped ensure that the website designs remained useful [72] to the Action Committee and CHAI and that we addressed any potential issues as they arose in the design process. We also emphasized continued and sustained collaboration [46] with CHAI as part of our strategy with the organization, Action Committee, and any other participants who wanted to remain engaged. Ahmed et al [46] used community-based participatory research methods to aim for “co-ownership and non-hierarchical, sustained collaboration between researchers and participants.” It was important to the HCI researchers on our team that CHAI, and particularly Action Committee members, felt ownership over the research and design processes, including paper authorship. Continued involvement in research beyond the initial interview or design session also demystifies the research process while letting participants and Action Committee members choose the level of continued involvement that is most comfortable for them, which may be none.

#### Community Access

From the start, we demonstrated a clearly defined vested interest between researchers, CHAI leadership, and the CHAI Action Committee. Harrington et al [14] discussed the importance of relationship building and vested interest between researchers and participants when accessing communities for participatory research. Our research collaboration began when CHAI leadership approached HCI researchers with the goal of redesigning the organization’s website. This directive came from the Action Committee, which identified a need for a more comprehensive and LGBTQ+-inclusive health website for young people in Washtenaw County and beyond. From the outset, the Action Committee’s goal became the goal of the project and informed design sessions, interviews, and follow-up activities. As the research collaboration was initiated by CHAI, CHAI’s vested interest was clearly demonstrated. Dreessen et al [73] argued for reciprocity in participatory design processes in which designers have a willingness to engage with communities and build relationships with them. Attendance to the Action Committee and CHAI meetings as well as our steps in designing the new website demonstrated this reciprocity.

Le Dantec and Fox [74] refer to community-based research’s “often invisible elements” that tend to occur even before the actual work. These elements include developing relationships, demonstrating commitment, and working together to overcome barriers [74]. The “invisible work” with CHAI and the Action Committee was vital to the overall design project. The community-based goals developed with CHAI and the Action Committee also helped with recruiting participants and establishing trust with them as our goals and outcomes were so clearly grounded in community needs. We could easily explain to participants in recruitment and throughout the interviews and design sessions that their suggestions would directly inform the new website. Processes such as user testing and frequent feedback sessions demonstrated to participants that we as researchers and developers were truthful in this claim when they could see how their participation and ideas were reflected in the new website. Our processes resembled more of an asset-based approach in which we centered a marginalized population’s “knowledge, strength, and capacities” [38] rather than simply their needs. Making these activities visible may be an important takeaway from this specific project for other participatory research.

#### Perceptions of Materials and Activities

In their reflections on participatory design activities, Harrington et al [14] carefully considered the specific activities and materials required. Accessibility of both the design activities and the required materials affects participant experiences and the resulting designs, especially when done entirely virtually. Owing to COVID-19, we conducted all interviews and design sessions remotely using videoconferencing, collaborative web-based documents, screen sharing, and other tools for eliciting the data needed. Although we initially considered the remote nature of the design sessions as a limitation, we soon learned that some aspects of the web-based workshops actually benefited participants and the research process. CHAI leadership and the researchers from the University of Michigan discussed options for remote collaboration to meet participants’ interests and comfort levels. In the design sessions, participants chose design tools (eg, digital drawing or drawing with pen and paper) that best suited their physical setup and preferred process. Remote design sessions provided freedom to explore different drawing and design options, allowing participants to choose whichever suited their needs and situation. The design sessions’ and interviews’ remote format generated a major concern: that participants might be in unsupportive home environments when asked to discuss information about gender and sexuality. We address this further in the following section.

Our findings are consistent with the argument by Harrington et al [14] that “brainstorming ‘blue sky’ ideas is a luxury practice that marginalizes those who have endured life with systemic disadvantage and resource scarcity.” Harrington and Dillahunt [75] both suggest and critique “futuring” as a possible design strategy with historically excluded populations to elicit more speculative results. In our work, the dystopian present heavily affected participants’ speculative designs [75]. The “dystopian present” for our participants is a United States in which access to transgender- and queer-inclusive health resources is scarce and dwindling in an increasingly politicized landscape. Some participants embraced more speculative approaches, such as suggesting an interactive map showing LGBTQ+ health care providers throughout the United States with verified reviews. This suggestion presents an ideal web resource but was unfortunately beyond the project’s capabilities. However, some “blue sky” ideas did inform website components, such as resources directing users to LGBTQ+ provider databases similar to the interactive map described. However, most participants’ design ideas for the website remained practical, meaning that participants focused their suggestions mainly on their lived experiences and information that was hard to find or nonexistent elsewhere. The designs remained bounded by practical possibilities for a website, seemingly based on participants’ past web-based experiences and on other web resources they had encountered. Participants perhaps kept the suggestions practical as they simply wanted their health information needs met by the CHAI website. However, aligned with Harrington and Dillahunt [75], it might be difficult to imagine “blue sky” ideas for a new web-based health resource when one’s most basic health needs are not met and are in fact actively under attack.

#### Unintentional Harm

As mentioned previously, the main unintentional harm we recognized in this research was the potential for endangering participants’ safety by possibly outing them through their participation. Harrington et al [14] discussed similar concerns with their participants regarding the disclosure of potentially sensitive information. In their reflection on participatory design and research, Duarte et al [76] similarly discussed the importance of creating safe spaces for participants from “vulnerable groups.” As researchers, we tried to ensure that both the interviews and design sessions functioned as safe spaces for participants to freely discuss LGBTQ+ health issues, potentially for one of the first times in a group with other LGBTQ+ young people. In this research, we feared potential harm from disclosure, particularly for participants aged <18 years. We also considered this even for older participants as many college-age participants were living with parents, guardians, or family because of COVID-19.

We received waivers of parental consent from our institutional review board to allow minors to participate without parental consent, which might unintentionally “out” them to parents or guardians. Not involving parents also ensured that we could reach LGBTQ+ young people who might especially struggle to find comprehensive and relevant health information because of unsupportive home environments. We particularly emphasized that participants could decline to answer any questions that might be uncomfortable in case they worried about disclosure while they were talking with us. The remote nature of all activities also required patience and flexibility when, for example, participants had to move to be in a more private location, such as a backyard or nearby park.

In collaboration with CHAI leadership, we accounted for possible harms in the research design process before recruitment and throughout the study so that we could best mitigate and reduce potential harm to participants. CHAI’s knowledge of community research and public health created an ongoing “disciplinary dialogue” [77] with HCI researchers to address harms. Although we could anticipate some harms as members of the LGBTQ+ community ourselves and from our past research with LGBTQ+ populations, we were better able to anticipate possible harm by relying on CHAI’s knowledge and extensive work with this specific community. Throughout the research process, adaptability based on participant needs, circumstances, and comfort was vital in our efforts to mitigate potential harms faced by participants. Beyond this LGBTQ+ health context, combining our experiences with the insights by Harrington et al [14], researchers can consider how to reach those within marginalized communities who might benefit the most from the products of HCI design activities while taking steps to ensure their safety and comfort.

#### Community Partner Leadership in HCI Research

We extend the work by Harrington et al [14] with the addition of community partner leadership as another tension and consideration for participatory HCI research with marginalized communities. A recent review of community-based research in HCI found that few studies brought in community partners in the problem definition stage, prioritized community knowledge, shared control with community partners, and involved community partners in analyzing data [78]. This places our study as an outlier in that our work was led by our community partner from the start and involved community members in all stages. Cooper et al [78] argued for structural change to align HCI research more with community-based principles, and Asad et al [79] suggested that researchers can function as “academic accomplices” in leveraging their resources to support the work of partners. We take these suggestions further and suggest that community partners can serve as leaders of research collaborations. Merkel et al [32] argued that participatory design should mean that community members lead the design process and maintain the infrastructure. This definition led our design process and website development. The project’s close connection with our partner informed how we handled each of the tensions discussed previously. However, this project moved beyond “community-based” in that our partner, CHAI, and community partner, the Action Committee, initiated the project and led its direction from the beginning to the final outcome. Beyond the community partner inclusion by Merkel et al [32], our community partner leadership consideration situates the community organization as the *main driving force behind the intended outcome of the design process*.

The LGBTQ+ young people who were members of the CHAI Action Committee clearly defined that a new website was their goal for the CHAI project. The HCI researchers on the team provided technical understanding and research and design knowledge to achieve the project’s goal, but CHAI brought this project to the HCI authors with an explicit final outcome in mind. In this sense, we had a final destination, and the non-CHAI members of our research team worked with CHAI to navigate the research and design process from identification and articulation of their need for a new website to the final website.

HCI researchers do not always need to initiate partnerships—sometimes, we can be more impactful as HCI researchers if we simply make ourselves available and wait for community partners to seek out our HCI expertise. HCI researchers wishing to engage in community-led research should consider how to signal their availability to the communities with which they wish to collaborate, such as by attending community events and meetings and getting involved in activism and advocacy. A community-led approach will not always align with publishing and conference deadlines as it involves substantial time and effort from HCI researchers; thus, it may, unfortunately, not be attainable for more junior academics who must worry about obtaining jobs and tenure. However, a community-led approach is powerful as it gives partners, especially those accustomed to the historically extractive nature of research relationships with marginalized communities, the opportunity and agency to seek out those partnerships if they so choose.

### New Design Recommendations for LGBTQ+ Digital Health Resources

In this section, we present design recommendations for future LGBTQ+ digital health resources that directly draw from what we learned in this study. Web-based health information resources must be designed differently for LGBTQ+ young people. Simply pasting a rainbow flag on a more general resource site is not sufficient, and in this paper, we have included evidence from many young people describing why. In this study, we see three primary hurdles for LGBTQ+ young people to effectively use health information on a resource website: (1) accessing information, (2) finding the right information, and (3) navigating identity disclosure concerns. We presented features to address each. First, building and deploying the new CHAI website provides vital access to LGBTQ+ health resources. Second, filters help site users locate the resources most useful to *them in particular*. Participants identified search features as important in finding information, yet being able to search may not always work, such as when young people do not know the terms to refer to the identity aspects they are exploring. Thus, filters can be an even more important way for LGBTQ+ young people to find useful resources, and participants also viewed filters as an intersectional mechanism for narrowing down resources. Third, a dark mode can help reduce LGBTQ+ identity disclosure in potentially unsafe settings. These results extend beyond previous participatory design work with LGBTQ+ and other marginalized populations [42-46,80] by providing specific design suggestions for how resource sites can best meet LGBTQ+ young people’s health information needs.

Throughout all the features we recommend, we found that LGBTQ+ young people require and desire resources tailored to their intersecting identities. Intersectionality informed the types of content LGBTQ+ young people mentioned wanting to see on the site, including resources, stories, and health experiences from people who shared their identities.

Throughout the Results section, we presented design considerations that participants wanted and did not want on the site. In this section, drawing from our results, we highlight 4 design recommendations—filters, dark mode, a reduced number of links to resources, and conscientious choice of graphics—with specific implications for future LGBTQ+ health resources. To demonstrate the need for these recommendations, we determined whether and how several current LGBTQ+ health resource sites use these recommended features. We surveyed the top 5 LGBTQ+ health resource sites from Google (as of July 2022), all from credible organizations, including Parents, Families, and Friends of Lesbians and Gays (PFLAG); the Centers for Disease Control and Prevention; and the Human Rights Campaign [81-85]. These 5 sites were the top 5 results in Google Search when querying the term “LGBTQ Health Resources” at the time of writing this paper. We do not intend for this to be a rigorous analysis but rather an exercise to see how certain features may or may not be currently deployed.

Most designs that participants sketched incorporated some type of *filtering* tool that included different identity components or areas of interest. Filtering may sound obvious, but aside from 1 state-level filter mechanism, none of the 5 LGBTQ+ resource sites evaluated included filters—yet we would argue that each would benefit from them. Filters related to identity would also better allow users to find resources for their specific overlapping identities and how they potentially affect one’s health. Material constraints and resource availability prevented our filtering from truly being “intersectional” as certain lived experiences are underrepresented in the final website. In the future, organizations with more resources can learn from our findings that LGBTQ+ young people desire health resources related to their intersectional identities and advanced filtering might better allow them to search and find relevant information to their own identities.

Next, using *dark mode* in site design is particularly useful for developing LGBTQ+ resources that users can browse without concern for unwanted identity disclosure. Of the 5 resource sites evaluated, none included dark mode functionality.

A *reduced number of resources* allows designers to direct their energy toward curating a few comprehensive, clear, and accurate resources rather than inundating users. The 5 resource sites evaluated provided an average of 112 links and a median of 14 links to resources (range 8-510). Although further research is needed to determine the optimal number of links, on the evaluated sites without filtering mechanisms and with limited search functionality, the number of links appeared overwhelming to the authors.

Finally, participants’ designs and feedback also stressed how important *conscientious use of images and graphics* is. Participants described viewing some health websites’ attempts to connect with potential users as pandering, insincere, or irrelevant. In total, 2 sites evaluated included no images, one included generic medical imagery, and one included an icon representing health. The remaining site included a photo of a diverse group of LGBTQ+ people. Future research could evaluate specific types of graphics and imagery more systematically with LGBTQ+ participants to determine which work best.

LGBTQ+ young people have particular design needs for health information resources, and we hope that, by highlighting these 4 underused design recommendations, we can influence future site design in LGBTQ+ contexts.

### Limitations

As with all research, this work involves several limitations. First, although we tried to include a diverse sample in our interviews and design sessions, transgender women, lesbians (though our sample did also include queer women who did not specifically identify as lesbians), Latinx people, and American Indian people were underrepresented in our participant group. Second, participants were more likely to attend or have attended college because of the composition of the Action Committee and our recruitment strategies. We attempted to mitigate these limitations and recruit a more diverse sample via our community partners and through additional web-based recruitment efforts. These limitations may have affected the results as we did not hear from some demographics. Specifically, centering underrepresented groups in future LGBTQ+ health research will be important to gain a fuller picture of health resource design. Third, this work is US-focused, which limits its capacity to apply to non-US and Western cultural contexts. Fourth, we chose not to ask participants about their home environments and parental approval of their LGBTQ+ identities. This information was only disclosed if patients chose to offer it when answering other questions. Finally, we lacked the resources needed to add personal stories from LGBTQ+ people, particularly focusing on intersectional identities and experiences, to the CHAI site. We leave this design suggestion for future work.

### Conclusions and Future Directions

LGBTQ+ young people have unique health information needs, and in this paper, we described our process of designing a web-based health resource with and for this population. We discuss several design features that can help LGBTQ+ young people access and find health resources that speak to them in particular, which can be especially useful for those at the intersection of several marginalized identities. These features include dark mode options, identity-based filtering, and conscientious graphics choices. We show how community-engaged and participatory approaches allow for new considerations for meeting marginalized populations’ health needs. Such interventions have the potential to improve marginalized people’s lives and reduce health disparities via increased access to resources, with the caveat that often HCI interventions cannot address the underlying systemic factors creating marginalization. Future researchers and designers can use and extend our findings and design processes, such as relying on community partner leadership, and further test some of the features we recommend to design and research future health resources for LGBTQ+ young people and other marginalized populations.

## Abbreviations

CHAI: Community Health Access Initiative
HCI: human-computer interaction
iCHECK-DH: Guidelines and Checklist for the Reporting on Digital Health Implementations
LGBTQ+: lesbian, gay, bisexual, transgender, queer, and questioning
PFLAG: Parents, Families, and Friends of Lesbians and Gays
RQ: research question
SRQR: Standards for Reporting Qualitative Research

## Data Availability

The data sets generated and analyzed during this study are not publicly available because of the content’s confidential nature relating to health and the participants’ marginalized identities but can be made available from the corresponding author upon reasonable request.
